# Mr. Cumming, on Old Ulcers

**Published:** 1801-08

**Authors:** Ralph Cuming

**Affiliations:** His Majesty's Ship Royal Oak, Portsmouth Harbour


### *To the Editors of the Medical and Phyſical Journal.*

Gentlemen,

As a further elucidation of the benefits ariſing from the uſe of ſtrips of adheſive plaſter in the cure of old and ill-conditioned Ulcers, I beg leave to publiſh, through the channel of your valuable Journal, the following caſe.

Margaret Allen, wife of one of the boatſwain's mates, made application to me ſoon after my appointment to the Royal Oak, for the cure of an ulcer of the above deſcription, on the ſuperior part of the malleolus internus of the right leg, about the ſize of a ſixpenny piece, and of a ſhape inclining to an oval, with hard and callous edges. On queſtioning her about its former treatment, ſhe ſaid it had been dreſſed very regularly with cerate or baſilicon, and was occaſionally touched with cuprum vitriolatum; and notwithſtanding all the care and attention which had been beſtowed upon it, for the ſpace of five months, it became more and more painful, and obſtinately reſiſted the plans that had been deviſed for its cure; and began to increaſe in ſize, which greatly added to the poor woman's uneaſineſs and apprehenſions for the ſafety of her ankle joint, ſuppoſing, from the pain ſhe conſtantly experienced, that the bone was diſeaſed. After applying the ſticking plaſters, firſt longitudinally, endeavouring to contract the face of the ſore, and then tranſverſly, by way of retaining the longitudinal ſtrips, and making more perfect preſſure, I retained the dreſſings by means of a ſoft compreſs of tow and an elaſtic flannel bandage, applied alternately round the arch of the foot and ankle; after which, in the courſe of two or three dreſſings, the whole of the calloſity diſappeared, and the ſore diſcharged a laudable pus, and was completely cicatrized in leſs than three weeks.

The

The beneficial effects of this mode of treatment are ſo immediate and ſtrikingly obvious as to excite both pleaſure and admiration, calling into action that deſire which medical men muſt ever feel to account for the modus operandi of every thing within the ſphere of their profeſſion, which I think may be explained in the following manner.

After an ulcer has remained long open, I conceive a great deal of atony exiſts in the part, which may have been produced by various cauſes; ſuch as irritation from ſubſtances applied to it, or probably from too great admiſſion of air, or the debilitating effects of unctuous matters, &c. all of which cauſes are perfectly obviated by proper and equable preſſure from the ſticking plaſter and roller, effecting a deſtruction of the old atonized irritable parts, and giving birth to new veſſels, vigorous, and fit for healthy granulation.

Whether the above account is ſatisfactory, I leave to the deciſion of my readers, or thoſe whoſe experience is greater than my own; however, this I may with truth affirm, that thoſe medical gentlemen who chuſe to diveſt themſelves of prejudice for old eſtabliſhed rules, will find their moſt ſanguine expectations fully gratified by a ſtrict adherence to this improved mode of dreſſing; and I think there will be no vanity in aſſerting that the community will be conſiderably benefited by it, for I myſelf have experienced its happy effects, long before either reading or hearing of them in the treatment of my patients on board of ſhips of war; but for want of that extenſive practice which falls to the lot of my brethren in hoſpitals, I never thought ſo ſeriouſly of its merits, as to give that decided preference it ſo juſtly deſerves, through diffidence in appearing before the public.

I am, &c.
RALPH CUMING.

*His Majesty's Ship Royal Oak,*

*Portsmouth Harbour, July* 17, 1801.

